# Post-stroke epilepsy in Saudi Arabia - frequency, management and outcomes at a comprehensive stroke center in Riyadh, Kingdom of Saudi Arabia

**DOI:** 10.1186/s12883-026-05091-1

**Published:** 2026-06-19

**Authors:** Meshari S. AlSudayri, Moath S. AlMosa, Sultan AlShehri, Ziyad AlGhweinem, Naif H. Ali, Ramesh Vishwakarma, Ismail A. Khatri

**Affiliations:** 1 Department of Neuroscience, Epilepsy Center, King Faisal Specialist Hospital & Research Centre (KFSH&RC), Riyadh, Saudi Arabia; 2 Department of Neurology, King Abdulaziz Medical City, MNGHA, PO Box 22490, Mail Code 1443, Riyadh, 11426 Kingdom of Saudi Arabia; 3 Department of Neurology, Neuroscience Centre, King Fahad Specialist Hospital, Dammam, Saudi Arabia; 4 Department of Internal Medicine, Najran University, Najran, Saudi Arabia; 5 Norwich Clinical Trial Unit, Norwich Medical School, University of East Anglia, Norwich, UK; 6 King Abdullah International Medical Research Center (KAIMRC), Riyadh, Saudi Arabia; 7 King Saud bin Abdulaziz University for Health Sciences, Riyadh, Saudi Arabia

**Keywords:** Post-stroke epilepsy, Frequency, management, Outcome, Saudi Arabia

## Abstract

**Background:**

Approximately 5% to 7% of patients with stroke develop post-stroke epilepsy (PSE). There is limited published literature about frequency, characteristics and outcomes of post-stroke epilepsy in Saudi population.

**Objectives:**

We determined the frequency, management and outcomes of post-stroke epilepsy in a Saudi cohort.

**Methods:**

After IRB approval, a retrospective chart-review was performed on patients admitted with stroke at King Abdulaziz Medical City, MNGHA, Riyadh between January 2016 and December 2020. PSE was defined as one or more seizures after the 7th day of incident stroke. The diagnosis was clinical, EEG was not required to make the diagnosis of PSE. Data was collected about demographic features, stroke characteristics, epilepsy management and outcomes. Data was analyzed using RStudio (R version 4.3.1.). Consecutive, nonrandom sampling technique was used and all patients were included from the study period.

**Results:**

A total of 2985 patients were included, of whom 2596 (87.0%) had ischemic stroke, 389 (13.0%) had hemorrhagic stroke. PSE occurred in 164 (5.49%) patients. The median age was 66.0 years (IQR:55.5–76.5), 102 (62.2%) were men, and 157 (95.7%) were Saudis. In ischemic group, 129 (4.97%) had PSE, whereas 35 (8.99%) in hemorrhagic group (*p* = 0.001). The commonest type of seizures was generalized in 96 (58.5%), focal in 45 (27.4%), unclassified in 23 (14.0%). Twenty-six (16%) patients had status epilepticus. Epileptiform abnormalities were seen in 16 (9.8%) on electroencephalogram. Treatment was started in 151 (93.8%) after first seizure, levetiracetam was commonest 1st antiseizure medication (ASM) used in 133 (82.6%). Side effects were reported in 11 (6.9%) patients; behavioral changes in 9 (5.5%), irritability in 6 (3.7%) patients. Nineteen (11.8%) patients needed 2nd ASM, 10 (6.1%) needed 3rd ASM. Median (IQR) follow-up was 26.0 (13.0–48.0) months. At last follow up, 120 (87%) were seizure free.

**Conclusions:**

Post-stroke epilepsy was common in our cohort. The patients with hemorrhagic stroke were more likely to have post-stroke epilepsy compared to ischemic stroke. Generalized seizures were the commonest type of seizure. Most patients had good control at last follow up requiring only monotherapy.

## Introduction

Stroke is among the most common causes of acquired epilepsy in the elderly population representing 45% of all newly diagnosed epilepsy cases after the age of 60 [1, 2]. Moreover, post-stroke epilepsy accounts for 11% of all adult epilepsy cases with some studies reporting prevalence as high as 21% [3, 4]. Post-stroke seizures (PSS) can be classified into two categories, early post-stroke seizures (or acute symptomatic seizures) if those occur within the first 7 days after the vascular event and late post-stroke seizures (or remote symptomatic seizures) if those happens after that time [5]. The importance of this classification stems from the differences in underlying mechanisms and risk of progression into epilepsy between early and late post-stroke seizures. Early post-stroke seizures usually result from the acute neuronal injury, glutamate release and the disruption of blood brain barrier. On the other hand, late post-stroke seizures are likely to occur due to the gliosis, chronic tissue scarring, neurodegeneration and alteration in synaptic plasticity [6]. International League Against Epilepsy (ILAE) defined epilepsy as two or more unprovoked seizures occurring at least 24 h apart or one unprovoked seizure with a 10 years risk of recurrence equal to the general population risk after 2 unprovoked seizures (60%) [7].

In Sweden, a retrospective cohort study was conducted on 240 patients with ischemic stroke and reported development of post-stroke epilepsy (PSE) in 13 patients with median time of onset of 237 days, 11 patients were started on levetiracetam (LVT), one patient was started on lamotrigine (LTG) and one patient on carbamazepine (CBZ). There was no data reported on seizure freedom, adverse effects and withdrawal from medication [8]. A meta-analysis of two randomized control trials, one comparing LVT with controlled release carbamazepine (CR-CBZ) and the other comparing LTG with CR-CBZ in the management of PSE reported no significant differences between either LVT or LTG and CR-CBZ in regard to seizure freedom. Adverse effects were lower in LVT and LTG groups in comparison to CR-CBZ. There was no difference in seizure freedom between LVT and LTG in indirect comparison, however, patients on LVT had more adverse effects than those on LTG (OR 6.87; 95% CI: 1.15–41.1) [4]. In one study in Korea, 3792 patients with ischemic stroke were reviewed and post-stroke seizures were identified in 124 (3.3%), 48 had early post-stroke seizures and 76 had late post-stroke seizures. Antiseizure medication (ASM) treatment effects were followed for a median follow-up duration of 26.0 months (IQR: 13.0–48.0). Of those who had early post-stroke seizure, 7 patients out of 48 were not started on ASMs and the recurrence rate of seizure was 43%. The rest of 41 patients were prescribed ASMs and their recurrence rate was 34%. Of those who had late post-stroke seizures, 7 patients out of 76 did not receive ASMs and they all (100%) had recurrence of seizure, 69 of the 76 patients with late post-stroke seizure were started on ASMs and only 43% had recurrent seizures [9].

In Saudi Arabia, there is limited published literature focusing on post-stroke epilepsy or its management. One single center study from Dammam reviewed the epidemiological profile of epilepsy patients, and reported 20 (11%) out of 184 patients to have post-stroke epilepsy [10]. Another study was conducted in Al-Qassem province which retrospectively studied 341 patients with epilepsy and found that 38 (11.7%) of those had post stroke epilepsy [11]. In a recent study from Saudi Arabia, where the patients were followed for 2 years, showed seizure recurrence in 38.6%. Females and patients on older ASM, as well as those with focal aware seizures were more likely to have seizure recurrence [12]. Another study of 1235 ischemic stroke patients from multiple centers in Saudi Arabia reported post-stroke seizures in 13.5% patients [13]. The authors did not differentiate between early and late PSS. Prior history of stroke, ICU admission and depression were associated with increased risk of PSS [13].

In this study we aimed to determine the frequency of post-stroke epilepsy in our cohort and assess the pattern of prescribing ASMs in our center, including the choice and time of starting ASMs. We also determined the efficacy, tolerability and adverse effects of various ASMs.

## Methods

This retrospective chart review was conducted on all patients who had a post-stroke seizure within the 5 years period between January 2016 to December 2020 at King Abdulaziz Medical City, Ministry of National Guard Health Affairs (MNGHA), Riyadh, Saudi Arabia. The study was approved by King Abdullah International Medical Research Center (KAIMRC)’s Institutional Review Board (NRC21R/220/05). Patient consent was waived due to retrospective nature of the study. No patient identifiers were included in data collection and analysis. The study adhered to the principles of the Declaration of Helsinki, 2013.

Post-stroke epilepsy was defined as one or more seizure that occurred among stroke patients after the first 7 days of incident stroke. The diagnosis of PSE was made on clinical grounds. Electroencephalogram (EEG) was obtained when feasible, but it was not considered mandatory for the diagnosis. As this was a retrospective study, the authors have no influence on the use of antiseizure medication (ASM). In our center, most clinicians prefer levetiracetam as first line antiseizure medication due to ease of administration and simpler titration. Primary outcome was defined as no recurrence of seizure up to 1 year of first medicine prescription, however, the seizure freedom was ascertained as various intervals including 6 months and last available follow up. For patients who did not follow up for one year or more, seizure freedom was determined at the last available visit. The seizure freedom was determined based on historical information from the patient or caregiver. Adverse effects were ascertained from patient reports or physician assessment as documented in medical records. Patients were divided into 3 groups; those who did not have seizure after stroke, those who had seizure within 7 days of stroke (early post-stroke seizure), and those who had seizure after 7 days of stroke (post-stroke epilepsy). Stroke severity was assessed based on National Institute of Health Stroke Scale (NIHSS) score, where score of 0–5 indicated mild stroke, 6–15 indicated moderate stroke, 16–24 indicated severe stroke, and 25 or more indicated very severe stroke. NIHSS is the is the most validated scale to determine the stroke severity [14]. It has not only been used in acute emergency room and in-patient settings but has also been shown to be reliable in assessing the stroke severity in population-based cohorts [14]. Stroke outcome was determined based on modified Rankin Scale score, where 0–2 indicated good outcome, 3–5 indicated poor outcome and 6 indicated death. Modified Rankin Scale, first introduced in 1957 by Dr. John Rankin was modified in 1980s and has evolved as the primary outcome measure for most acute stroke trials [15].

Clinical, radiological and outcome data was collected in a pre-specified data collection form. Statistical analysis was performed using SAS software version 9.4. Categorial variables were presented as frequencies and percentages, while continuous variables were summarized using median and interquartile range (IQR) after assessing normality using the Shapiro-Wilk test. Comparisons between groups were performed using appropriate inferential methods based on the types and distribution of data. Continuous variables were compared using the Wilcoxon rank-sum test. Categorial variables were analyzed using the Pearson’s Chi-squared test or the Fisher’s exact test when expected cell counts were small. No transformations or modifications were applied to the original data. Missing data were reported, and analyses were conducted based on available cases. All statistical tests were two-tailed, and a p-value < 0.05 was considered statistically significant.

## Results

A total of 2985 stroke patients were admitted during the study period, of whom 2596 (87.0%) had ischemic stroke, whereas 389 (13.0%) had hemorrhagic stroke. Poststroke epilepsy (PSE) occurred in 164 (5.49%) of all patients. Among the 164 patients, 129 (78.7%) had ischemic stroke, whereas 35 (21.3%) had hemorrhagic stroke. In the ischemic stroke group, 129/2596 patients (4.97%) had PSE, whereas 35/389 (8.99%) of all hemorrhagic stroke patients had epilepsy (*p* = 0.001). Among the 164 patients with PSE, most patients 119 (72.6%) had first stroke whereas 46 (28.9%) patients were admitted with recurrent stroke. Among the 164 patients with PSE, 102 patients (62.2%) were men, 157 (95.7%) were Saudis, 145 (88.4%) were married, and only 20 (13.3%) were currently employed at the time of stroke. The median age of patients with PSE was 66.0 years (IQR: 55.5–76.5), with no significant difference between males and females in terms of age (*p* = 0.433). The median BMI of patients with PSE was 26.0 kg/m² (IQR: 22.0–30.5). Women had a significantly higher BMI compared to men (30.0 [26.0–35.0] vs. 24.0 [21.0–28.0], *p* < 0.001; Table 1).

**Table 1:** Demographic characteristics of patients with post-stroke epilepsy

Characteristic	*N* = 164	*p*-value
Gender		
Male	102 (62.2%)	
Female	62 (37.8%)	
Nationality		
Saudi	157 (95.7%)	
Non-Saudi	7 (4.3%)	
Age (in years)	66.0 (55.5–76.5)	
Age (males)	66.0 (55.0–75.0)	
Age (females)	69.0 (58.0–77.0)	0.433
Age (Saudi)	67.0 (56.0–77.0)	
Age (Non-Saudi)	58.0 (53.0–62.0)	
Height (cm)	161.0 (154.0–168.0)	
Weight (kg)	68.0 (58.4–79.0)	
BMI	26.0 (22.0–30.5)	
BMI (males)	24.0 (21.0–28.0)	< 0.001
BMI (females)	30.0 (26.0–35.0)	
Marital status		
Married	145 (88.4%)	
Single	9 (5.5%)	
Widow	7 (4.3%)	
Divorced	3 (1.8%)	
Occupation*		
Employed	20 (13.3%)	
Unemployed	85 (56.7%)	
Retired	15 (10.0%)	
Housewife	28 (18.7%)	
Student	2 (1.3%)	

*The variable had 14 mission records

**Fig. 1:**
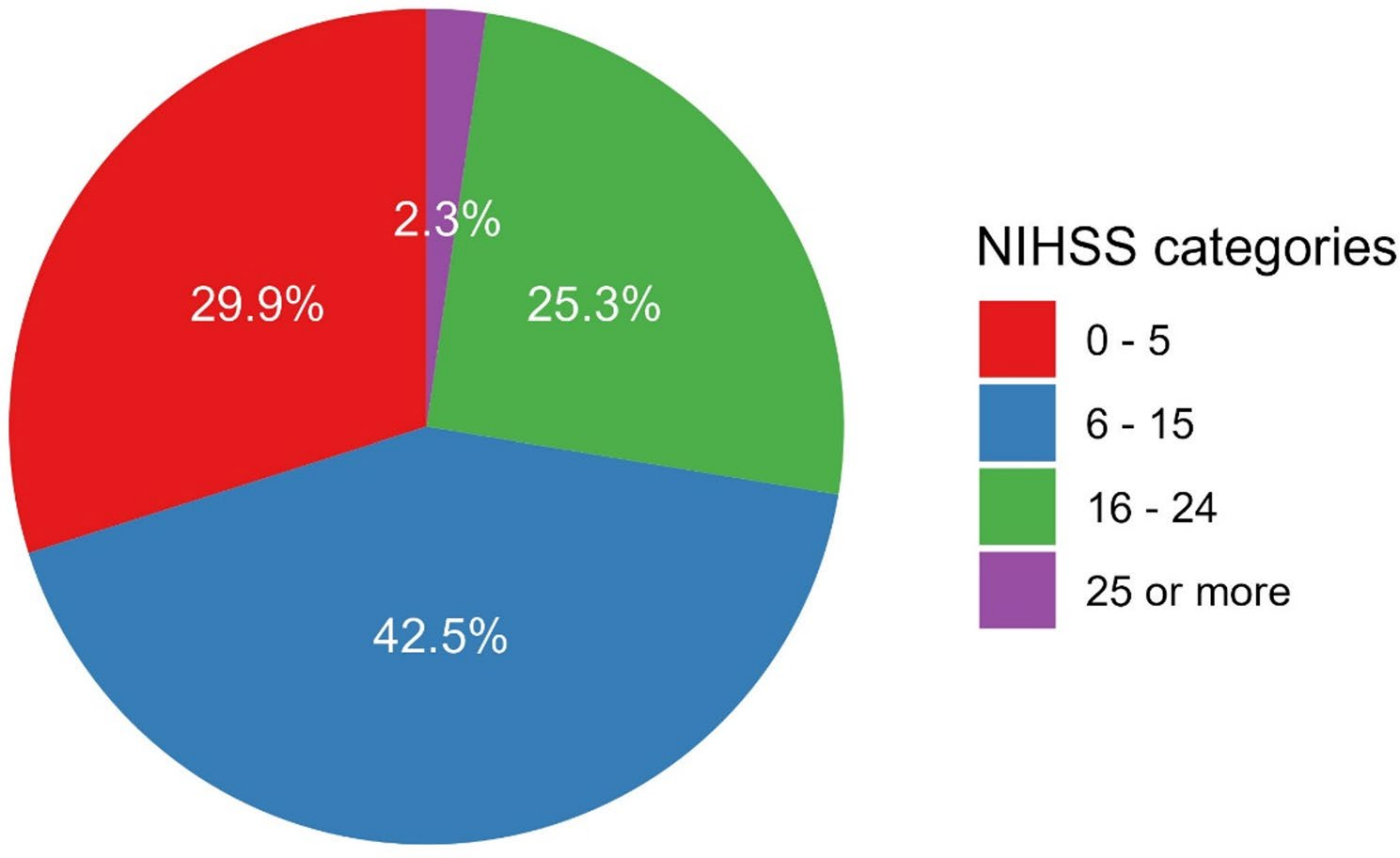
Stroke severity at the time of presentation based on NIHSS score

Predominant symptoms among the 164 patients with PSE included unilateral weakness in 113 patients (68.9%), change in speech in 90 (54.9%) patients and unilateral numbness in 51 patients (31.1%). Notably, altered level of consciousness at presentation was observed in 59 patients (36.0%), whereas seizure at onset of stroke was reported in 21 (12.8%) patients. Approximately a third of patients had normal blood pressure at presentation (32.5%). The median duration of hospital stay for patients with PSE was 19.0 days (IQR:8.0–52.0), with 65 (39.9%) patients staying between 7 to < 30 days. Initial NIHSS score among the 164 patients with PSE was 0 to 5 among 26 patients (29.9%), 6 to 15 among 37 patients (42.5%), 16 to 24 among 22 patients (25.3%) and 25 or more among two patients (2.3%, Fig. 1). A large number of patients did not have NIHSS documentation at the time of presentation.

Among the 164 patients with PSE, ischemic stroke was the most common type, accounting for 129 cases (78.7%), followed by hemorrhagic stroke in 35 cases (21.3%). The majority of strokes were first-time occurrences, with 119 cases (72.6%), while 46 cases (28.9%) were recurrent strokes. Neuroimaging findings for ischemic strokes revealed the left middle cerebral artery (Lt MCA) as the most affected site, with 30 cases (24.2%), followed by the right MCA with 28 cases (22.6%). For hemorrhagic strokes, neuroimaging showed involvement of various regions, with the left basal ganglia (Lt BG) and right lobar regions each accounting for 8 cases (20.5%) as the most common sites. The TOAST classification system for the patients with ischemic stroke indicated that large artery atherosclerosis was the leading cause of ischemic strokes, identified in 54 cases (32.9%), followed by cardioembolism in 37 cases (22.6%, Table 2). There was no significant difference between various clinical characteristics and outcomes when right hemispheric strokes were compared to the left (data available but not presented).

**Table 2:** Characteristics of stroke

Characteristic	*N* = 164	Missing
Type of stroke		0 (0.0%)
Ischemic stroke	129 (78.7%)	
Hemorrhagic stroke	35 (21.3%)	
1st stroke	119 (72.6%)	0 (0.0%)
Recurrent Stroke	46 (28.9%)	5 (3.0%)
Neuroimaging (for ischemic strokes)		
Right Middle Cerebral Artery (MCA)	28 (22.6%)	
Right Anterior Cerebral Artery (ACA)	7 (5.6%)	
Right Posterior Cerebral Artery (PCA)	11 (8.9%)	
Left Middle Cerebral Artery (MCA)	30 (24.2%)	
Left Anterior Cerebral Artery (ACA)	2 (1.6%)	
Left Posterior Cerebral Artery (PCA)	8 (6.5%)	
Bilateral hemispheric infarctions	3 (2.4%)	
Multifocal infarctions	13 (10.5%)	
Other locations	22 (17.6%)	
Neuroimaging (for hemorrhagic strokes)		
Right basal ganglia (BG)	5 (12.8%)	
Right lobar	8 (20.5%)	
Right thalamic hemorrhage	1 (2.6%)	
Left basal ganglia (BG)	8 (20.5%)	
Left lobar	5 (12.8%)	
Left thalamic hemorrhage	4 (10.3%)	
Multifocal hemorrhage	5 (12.8%)	
TOAST classification		0 (0.0%)
Large artery atherosclerosis	54 (32.9%)	
Small vessel disease	11 (6.7%)	
Cardioembolism	37 (22.6%)	
Stroke of other determined etiology	8 (4.9%)	
Stroke of undetermined etiology	17 (10.4%)	

Among the 164 patients with post-stroke epilepsy, 32 (20.0%) also had seizure within first 7 days of incident stroke. The majority of seizures were of the generalized type (58.5%), followed by focal seizures (27.4%). Status epilepticus was observed in 26 cases (16.0%). Electroencephalogram (EEG) was performed in 88 (54%) of the patients, that showed epileptiform discharges or seizures in 16 cases (9.8%), while 75 cases (46.0%) had no EEG performed. ASMs were used in 162 patients (99.4%), with initiation after the first unprovoked seizure in the majority of cases (93.8%) (Table 3).

**Table 3:** Characteristics of seizures and ASMs use

Characteristic	*N* = 164	Missing
Seizure in the first 7 days	32 (20.0%)	4 (2.4%)
Seizure after the 1st 7 days	164 (100.0%)	0 (0.0%
Type of seizure		0 (0.0%)
Generalized	96 (58.5%)	
Focal	45 (27.4%)	
Not classified	23 (14.0%)	
Status epilepticus	26 (16.0%)	
EEG		1 (0.6%)
No epileptiform discharges or seizures	72 (44.2%)	
Epileptiform discharges or seizures	16 (9.8%)	
Not done	75 (46.0%)	
Antiseizure medications (ASMs) use	162 (98.8%)	1 (0.6%)
Time of starting ASMs		3 (1.8%)
After 1st unprovoked seizure	151 (93.8%)	
After 2nd unprovoked seizure	10 (6.2%)	

The most commonly prescribed first ASM was levetiracetam, with 133 patients (82.6%) receiving it, followed by phenytoin in 15 patients (9.3%). Side effects after the first ASM were reported in 11 patients (6.9%), with the most common being behavioral changes in 6 patients (54.5%). Discontinuation of the first ASM occurred in 5 patients (45.5%), while 7 patients (63.6%) switched to another ASM. Reasons for discontinuation or switching included side effects and therapy failure. Adding a second ASM was necessary for 37 patients (22.5%), with levetiracetam and valproic acid being the most commonly added medications (*n* = 12, 32.4% for each).

Additionally, 10 patients (32.3%) required the addition of a third ASM (Table 4). When the choice of ASM was assessed based on stroke severity (NIHSS score 15 or less and > 15), levetiracetam was the only first line ASM used in patients with NIHSS of > 15, whereas multiple first line ASMs were used in patients with mild or moderate strokes (*p* = 0.008). Data available, but not shown.

**Table 4:** Pattern of the antiseizure medications (ASMs) utilization and adverse events

Characteristic	*N* (%)	Missing
1st ASM		3 (1.8%)
Levetiracetam	133 (82.6%)	
Valproic acid	8 (5.0%)	
Carbamazepine	3 (1.9%)	
Carbamazepine (Controlled release)	1 (0.6%)	
Phenytoin	15 (9.3%)	
Lamotrigine	1 (0.6%)	
Lacosamide	0 (0.0%)	
Side effect after 1st ASM	11 (6.9%)	4 (2.4%)
Behavioral change	6 (54.5%)	
Hyponatremia	0 (0.0%)	
Skin rash	0 (0.0%)	
Irritability	5 (45.5%)	
Tremor	1 (9.1%)	
Dizziness	0 (0.0%)	
Discontinuation of 1st ASM	5 (45.5%)	0 (0.0%)
Switching to another ASM	7 (63.6%)	0 (0.0%)
Reason of discontinuation of the 1st ASM, switching or adding another AED		3 (27.3%)
Failure of therapy	1 (12.5%)	
Side affects	4 (50.0%)	
Both	3 (37.5%)	
No more seizures	0 (0.0%)	
Adding 2nd ASM	37 (22.5%)	4 (2.4%)
2nd ASM		
Levetiracetam	12 (32.4%)	
Valproic acid	12 (32.4%)	
Carbamazepine	2 (5.4%)	
Phenytoin	5 (13.5%)	
Lamotrigine	2 (5.4%)	
Lacosamide	4 (10.8%)	
Discontinuation of 2nd ASM	4 (12.9%)	
Adding 3rd ASM	10 (32.3%)	

Compliance with ASM therapy was observed in 138 patients (91.4%). Recurrent seizures within 6 months (data available on 65 patients) and 1 year (data available on 57 patients) after starting ASM therapy were reported in 49 (33.1%) and 35 (24.6%) patients respectively. The median (IQR) duration of the last follow-up visit from the incident stroke was 26.0 months (13.0–48.0). At the last follow-up (data available for 146 patients), 120 patients (87.0%) were seizure-free. When various types of seizures (generalized, focal or unclassified) were analyzed in relation to ASMs, there was no difference in the need for addition of 2nd ASM (*p* = 0.071), recurrence of seizures at 1 year (*p* = 0.392), or seizure freedom at last follow up (*p* = 0.071). Depending on the type of 1st ASM and need for 2nd ASM (*p* = 0.310), recurrent seizure at one year (*p* = 0.681) and seizure freedom at last follow-up (p = > 0.999), there was no difference between various ASMs (data available, but not presented).

Stroke related complications among 164 patients with PSE included urinary tract infection (UTI) in 35 patients (21.5%), pneumonia in 22 patients (13.5%), and recurrent stroke in 21 patients (12.9%), whereas 93 patients (60.8%) experienced no complications. Majority of patients 143 (88.3%) were discharged home, whereas 10 (6.2%) of the patients died. Discharge and long-term stroke outcome were determined based on modified Rankin Scale score which was unfortunately missing in one-sixth of the patients at discharge, and almost one-third of patients at the last follow up (Table 5).

**Table 5:** Medication compliance and various outcomes

Characteristic	*N* = 164	Missing
Compliance to ASMs	138 (91.4%)	13 (7.9%)
Recurrent seizure in 6 months after starting ASM	49 (33.1%)	16 (9.8%)
Recurrent seizure in 1 year after starting ASM	35 (24.6%)	22 (13.4%)
Last follow up visit from incident stroke (in months)*	32.7 ± 31.4	45 (27.4)
Seizure free at last follow up	120 (87.0%)	26 (15.9%)
Complications		
Recurrent stroke	21 (12.9%)	1 (0.6%)
Pneumonia	22 (13.5%)	1 (0.6%)
Urinary tract infection	35 (21.5%)	1 (0.6%)
Depression	12 (7.4%)	1 (0.6%)
Fall	5 (3.1%)	2 (1.2%)
Sepsis	19 (11.7%)	1 (0.6%)
No complication	93 (60.8%)	11 (6.7%)
Discharge disposition		2 (1.2%)
Discharge home	143 (88.3%)	
Discharge to inpatient rehabilitation	1 (0.6%)	
Discharge to long term facility/nursing home	5 (3.1%)	
Transfer to another hospital	3 (1.9%)	
Death	10 (6.2%)	
MRS at discharge		26 (15.9%)
0–2	32 (23.2%)	
3–5	103 (74.6%)	
6	3 (2.2%)	
MRS at last follow up		63 (38.4%)
0–2	27 (26.7%)	
3–5	74 (73.3%)	
6	0 (0.0%)	

*Median (Q1 to Q3)

Post-stroke epilepsy was more common in patients with hemorrhagic strokes compared to ischemic strokes (9.0% vs. 4.97%; *p* = 0.001). Significantly higher proportions of patients with hemorrhagic stroke experienced seizures within the first 7 days compared to those with ischemic stroke (34.3% vs. 16.0%; *p* = 0.029). There was no significant difference in the frequency of status epilepticus between the patients with ischemic stroke and hemorrhagic stroke (14.1% vs. 22.9%; *p* = 0.205). There was no difference in seizure freedom at last follow up (*p* = 0.365), stroke related complications (*p* = 0.075), mRS at discharge (*p* = 0.133) and death (*p* = 0.691) between the two groups (Table 6).

**Table 6:** Comparison of various clinical characteristics and outcomes based on type of stroke

Characteristic	Ischemic stroke	Hemorrhagic stroke	*p*-value
Frequency of PSE	129/2596 (4.97%)	35/389 (9.0%)	0.001
Initial NIHSS	10.0 (5.0–16.0)	13.5 (8.0–16.0)	0.193*
Seizure in the first 7 days	20 (16.0%)	12 (34.3%)	0.029
Status epilepticus	18 (14.1%)	8 (22.9%)	0.205
1st ASM			
Levetiracetam	104 (82.5%)	29 (82.9%)	> 0.999
Compliance	108 (90.8%)	30 (93.8%)	0.736
Seizure free at last follow up	97 (88.2%)	23 (82.1%)	0.365
No complication	77 (64.7%)	16 (47.1%)	0.075
Discharge disposition			
Death	9 (7.1%)	1 (2.9%)	0.691
Discharge home	114 (89.8%)	29 (82.9%)	0.251
mRS at discharge	4.0 (2.0–4.0)	4.0 (3.0–4.0)	0.133*

*Wilcoxon rank sum test; otherwise, Pearson’s Chi-squared test or a Fisher’s exact test were applied as appropriate

When the characteristics and outcomes of 164 patients with PSE were compared based on gender, the only significant difference was higher frequency of status epilepticus in males (20.8% vs. 8.1%; *p* = 0.046). There was no significant difference in stroke severity (*p* = 0.877), discharge mRS (*p* = 0.487) seizure freedom at last follow up (p = > 0.999) or death (*p* = 0.508) between the two gender.

When the 164 patients with PSE were divided according to the age in groups of age 60 or under and age more than 60 years, the only significant difference was in discharge mRS which was less in the younger age group (*p* = 0.004). There was no significant difference in the stroke severity (*p* = 0.679), frequency of status epilepticus (*p* = 0.374), seizure freedom at last follow up (*p* = 0.112), or death (*p* = 0.496) between those younger than 60 years of age or more than 60 years.

## Discussion

In this study, we evaluated the frequency, management, and outcomes of post-stroke epilepsy (PSE), focusing on the choice and timing of antiseizure medication (ASM) initiation, as well as efficacy and tolerability of treatments. The incidence of PSE has been reported to be approximately 10%, with early post-stroke seizures occurring in about 7% of patients; the risk is highest within the first year after stroke, and up to 84% of cases develop within the first two years of the cerebrovascular event [16]. The diagnosis of PSE was mostly clinical and when possible, supported by electroencephalographic data. However, EEGs were performed in just a little over 50% of patients with PSE.

Our results indicate that PSE occurred in 5.49% of all stroke patients, with a higher incidence in hemorrhagic stroke patients (8.99%) compared to ischemic stroke patients (4.97%). These findings align with studies that have reported varying incidences of PSE, often influenced by stroke type and patient demographics [1, 16]. A recent study from Saudi Arabia reported post-stroke seizures in 13.5% of ischemic stroke patients. This study did not include hemorrhagic stroke and did not differentiate between early PSS and late PSS [13].

A retrospective cohort study conducted in Sweden observed post-stroke epilepsy in 13 patients out of 240 ischemic stroke patients, with a median onset of 237 days. While the incidence in our study was higher, the time of onset for PSE (measured from the first seizure) in our cohort showed a more immediate post-stroke pattern, likely due to different study designs and patient populations [8].

The underlying mechanisms of epileptogenesis after stroke are multifactorial, involving maladaptive synaptic plasticity, gliotic scarring and persistent hyperexcitability of cortical networks. Blood–brain barrier disruption and the release of blood breakdown products, such as hemosiderin and albumin, trigger oxidative stress, Transforming Growth Factor beta (TGF-β) mediated astrocytic activation, glutamate excitotoxicity, and neuroinflammatory cascades, all of which contribute to the transformation of acute symptomatic seizures into chronic epilepsy [17].

The frequency of early post-stroke seizures (within 7 days) in our cohort was notably higher in hemorrhagic stroke patients compared to ischemic stroke patients (34.3% vs. 16.0%), which is consistent with the findings from the study by Kim et al., who reported a higher incidence of early seizures in hemorrhagic stroke patients [9]. This supports the idea that hemorrhagic strokes may cause more acute neuronal injury, leading to a higher risk of early-onset seizures. Conversely, ischemic stroke may involve a more gradual pathophysiological process, contributing to later seizure development. These findings highlight the importance of considering stroke type when evaluating the risk of seizures and determining the appropriate timing for ASM initiation.

As our study was retrospective in nature, the authors had no role in selecting antiseizure medication for specific patients based on their stroke type, or seizure classification. Levetiracetam (LVT) was the most commonly prescribed ASM in our cohort, consistent with the findings from the meta-analysis by Brigo et al., which suggested that LVT is frequently used for PSE management due to its favorable side effect profile compared to older ASMs like carbamazepine and phenytoin [4]. This is further corroborated by a study from Korea, where LVT was found to be effective in reducing recurrence rates in post-stroke seizures, particularly when compared with other ASMs like carbamazepine [9]. We could not systematically compare the efficacy of levetiracetam over other ASMs. In our cohort, 93.8% of patients who experienced seizures were started on ASMs after their first event, with 82.6% receiving LVT as their primary treatment, demonstrating its preference in our local clinical practice. The recent data also demonstrated comparable efficacy of LVT to carbamazepine, but with significantly fewer adverse effects, supporting the shift toward LVT use in modern clinical practice [18].

Our results also highlight that a significant proportion of patients (22.5%) required a second ASM due to recurrent seizures, and 6.1% required a third ASM. This suggests that despite the initial efficacy of LVT, it may not be sufficient for all patients, reflecting a common clinical challenge in managing post-stroke epilepsy. This mirrors the findings of the study by Kim et al., where some patients required additional ASM therapy to control seizures, despite initial treatment [9]. The incidence of adverse effects in our cohort (6.9%) was relatively low, with behavioral changes and irritability being the most commonly reported side effects, which is consistent with the side effects profile seen in other studies evaluating ASMs like LVT and carbamazepine.

Interestingly, the study found no significant differences in seizure freedom between ischemic and hemorrhagic stroke patients at 6 months and 1 year, which is somewhat contradictory to findings from other studies suggesting worse outcomes in hemorrhagic stroke patients [12]. This could be due to the relatively small sample size of hemorrhagic stroke patients in our cohort (35 out of 164 PSE cases), which may limit the ability to detect differences in long-term outcomes. The follow up data at 6 months and 1 year was available only on less than 50% patients, which may be another contributor to this finding. Additionally, the high rate of seizure freedom at 1 year (78.7%) across all stroke types in our cohort suggests that with timely and appropriate treatment, post-stroke epilepsy can be effectively managed in the majority of patients, regardless of stroke subtype. This is in concordance with recent reviews that suggest treatment approaches and expected seizure control are generally the same for both stroke types [19]. Our findings may have been affected by a large number of patients that were lost to follow up at 1 year. A recent study from Saudi Arabia reported seizure recurrence rate of 36.8% at 2 years, which is much higher [12]. This study found female gender, use of older ASMs, focal aware seizures and interictal epileptiform abnormalities to be predictors of seizure recurrence. In our cohort, the clinical outcome data in terms of NIHSS and mRS was missing for a significant proportion of patients at last follow up, some of the correlations may have been affected by this missing data.

When comparing the outcomes of patients under and over 60 years old, our findings align with previous research indicating that age may not be a significant factor in PSE outcomes, as no significant differences were found in the frequency of seizures or seizure freedom rates. This is in agreement with a study by Forsgren et al., which did not report a significant age-related impact on the development of unprovoked seizures following stroke [2]. A recent hospital-based prospective study also found no significant association between age and PSE outcomes [1]. However, it remains essential to continue investigating the role of age and other demographic factors in influencing PSE risk and treatment outcomes.

One of the strengths of our study is that a large number of stroke patients (nearly 3000) were included in consecutive fashion over 5 years of study. We were able to identify the type of stroke and mechanism of stroke in most cases. The type and timing of ASM use was also available for most patients. In general, the follow up was available up to almost 3 years which is a good duration to assess the long-term outcome of PSE.

One key limitation of our study is its retrospective nature, which may introduce biases related to the completeness of patient medical files, especially regarding medication compliance, adverse effects and follow-up data. Initial NIHSS, discharge mRS and long term follow up mRS was missing on considerable number of patients. A large number of patients did not follow up at 3 month and 1-year intervals. Nearly half of the patients did not get EEG, and the diagnosis of PSE in most cases was based on clinical event. These missing data at the follow up, limit the real determination of clinical outcome as well as PSE outcome. Performance on EEG although very relevant, is not required to make the diagnosis of epilepsy in appropriate clinical context. Additionally, the interictal EEG may not show epileptiform activities, as seen in majority of our patients who had EEG study. Unfortunately, long term stroke related outcomes were missing for many patients which although do not direct impact the objectives of our study, may have provided additional information.

## Conclusions

In conclusion, in our cohort of Saudi stroke patients, post-stroke epilepsy (PSE) is a significant complication following both hemorrhagic and ischemic strokes, with a higher incidence observed in hemorrhagic strokes. Levetiracetam was the most prescribed antiseizure medication (ASM) and demonstrated favorable efficacy and tolerability profiles. A subset of patients required multiple ASMs due to recurrent seizures, highlighting the complexity of managing PSE. The overall seizure freedom rate at 1 year was high, suggesting that with appropriate and timely ASM initiation, PSE can be effectively controlled in most patients. These findings highlight the importance of early identification, individualized treatment strategies, and continued monitoring for optimal management of post-stroke epilepsy.

Further research, including prospective multicenter studies are required to enhance treatment protocols and better understand long-term outcomes in diverse patient populations.

## Data Availability

All the data is available with the corresponding author and can be provided upon request after permission from the institutional review board.
